# Cancer-on-chip: a breakthrough organ-on-a-chip technology in cancer cell modeling

**DOI:** 10.1007/s11517-024-03199-5

**Published:** 2024-10-14

**Authors:** Babak Nejati, Reza Shahhosseini, Mobasher Hajiabbasi, Nastaran Safavi Ardabili, Kosar Bagtashi Baktash, Vahid Alivirdiloo, Sadegh Moradi, Mohammadreza Farhadi Rad, Fatemeh Rahimi, Marzieh Ramezani Farani, Farhood Ghazi, Ahmad Mobed, Iraj Alipourfard

**Affiliations:** 1 Liver and Gastrointestinal Disease Research Center, Tabriz University of Medical Sciences, Tabriz, Iran; 2 Faculty of Medicine, Istanbul Medipol University, Istanbul, Turkey; 3 Islamic Azad University of Tonekabon, Tonekabon, Iran; 4 Department of Midwifery, Ardabil Branch, Islamic Azad University, Ardabil, Iran; 5 Faculty of Medicine, Tabriz University of Medical Sciences, Tabriz, Iran; 6 Ramsar Campus, Mazandaran University of Medical Sciences, Ramsar, Iran; 7 Department of Immunology, Faculty of Medicine, Ahvaz Jundishapur University of Medical Sciences, Ahvaz, Iran; 8 Faculty of Medicine, Yazd University of Medical Sciences, Yazd, Iran; 9 Division of Clinical Laboratory, Zahra Mardani Azar Children Training Research and Treatment Center, Tabriz University of Medical Sciences, Tabriz, Iran; 10 Faculty of Allied Medicine, Shahid Beheshti University of Medical Sciences, Tehran, Iran; 11 Department of Community Medicine, Faculty of Medicine, Social Determinants of Health Research Center, Tabriz University of Medical Sciences, Tabriz, Iran; 12 Institute of Physical Chemistry, Polish Academy of Sciences, Marcina Kasprzaka 44/52, 01-224 Warsaw, Poland

**Keywords:** Organ-on-a-chip (OoC), Tumor microenvironment, Cancer cell modeling, Cancer on-chip, Microfluidics, Bioprinting, 2D/3D culture

## Abstract

Cancer remains one of the leading causes of death worldwide. The unclear molecular mechanisms and complex in vivo microenvironment of tumors make it difficult to clarify the nature of cancer and develop effective treatments. Therefore, the development of new methods to effectively treat cancer is urgently needed and of great importance. Organ-on-a-chip (OoC) systems could be the breakthrough technology sought by the pharmaceutical industry to address ever-increasing research and development costs. The past decade has seen significant advances in the spatial modeling of cancer therapeutics related to OoC technology, improving physiological exposition criteria. This article aims to summarize the latest achievements and research results of cancer cell treatment simulated in a 3D microenvironment using OoC technology. To this end, we will first discuss the OoC system in detail and then demonstrate the latest findings of the cancer cell treatment study by Ooc and how this technique can potentially optimize better modeling of the tumor. The prospects of OoC systems in the treatment of cancer cells and their advantages and limitations are also among the other points discussed in this study.

**Graphical Abstract:**

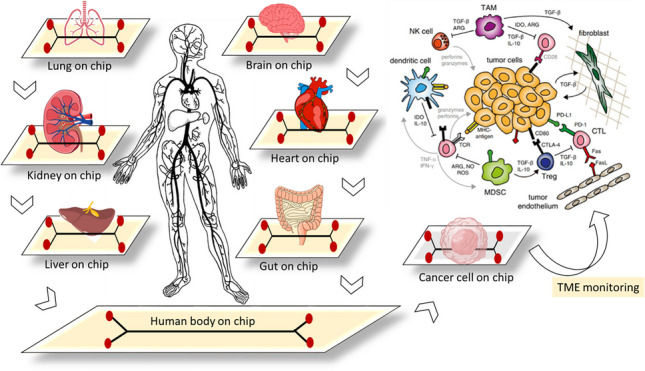

## Introduction

Cancer remains a leading cause of death worldwide, and cancer treatment places a significant burden on health care [1, 2]. Despite the high mortality rate from cancer, significant advancements have been made over decades in research, leading to the development of targeted therapies, immunotherapies and combination therapies. Chemotherapy is well-known as an effective treatment for many types of cancer. Unfortunately, it was not as efficient as expected in eradicating all cancer cells. The mechanism of this failure is still not fully understood [3]. Surgery of solid tumors is one of the most effective ways to treat cancer, which, unfortunately, due to the low prognosis, the failure to distinguish between healthy tissue and cancerous tissue by visual observation and touch during surgery greatly reduces the level of satisfaction [4, 5]. In other words, diagnosis of incorrect tumor margins as well as small tumors (for example, less than 1 mm) during surgery leads to excessive removal of normal and healthy tissues, which will have irreparable complications for the patient [4, 5]. However, existing cancer treatments have their own side effects, and the abnormal extracellular matrix (ECM) of solid tumors resists therapeutic components and immune cell invasion. Therefore, recent efforts have been made to develop anticancer approaches to address these limitations [1, 2]. Nanomaterials (NMs) are considered useful in oncology because their small size provides sensitive cancer detection ability and effective treatment results [6, 7]. NM-based therapies have many advantages over conventional chemotherapy, including increased bioavailability, dose-responsiveness, and targeting efficiency with fewer side effects [6, 7]. Nanoparticles can provide photothermal and photodynamic therapy for ROS overproduction and hyperthermia for cancer suppression [8]. Although various treatments are being considered for the treatment of cancer, the prognosis remains poor. According to the consensus that exists in the scientific community, the current models used in the initial tests of new diagnostic and therapeutic techniques do not correctly represent the tissue microenvironment in humans [9, 10]. For example, the most common in vivo models that can be compared to humans are transgenic mice that are immunodeficient and, depending on the assay, have specific gene mutations, which can bias the response compared to humans. Despite some suitable features, such as similarities in disease manifestations, these models are not able to clarify some molecular mechanisms, such as the interaction of individual cells, including pathogenic cells, organ cells, and immune cells in the physiological environment [9, 10]. In any case, the development of modern methods to overcome these limitations is necessary and unavoidable. In this regard, one of the most interesting techniques in cancer management is the “organ-on-chip (OoC)” method, which has attracted the attention of researchers in the last few years. OoC technology creates microenvironment simulations by combining two or more cell types. Combining organoids and organ chips offers extensive research opportunities for accurate tumor treatments [11, 12]. OoC technology’s main benefit lies in its ability to replicate the structural and functional complexity of living tissues and organs, unlike in vitro cell culture methods, which cannot reproduce dynamic mechanical and biochemical microenvironments [11]. The difficulty is not only in recreating the specific traits of organs but also in simulating how they interact dynamically, the transportation processes, and how they react to stimuli [13, 14]. Moreover, OoC models, which are based on microfluidic chips containing chambers for 3D tumor-cell culture, allow us to establish a regulated tumor microenvironment (TME). Consequently, OoC models are increasingly utilized to methodically examine the impact of the TME on the different stages of cancer metastasis [13, 14]. The focus of this review is to provide an overview of OoC developed for the treatment of different types of cancer and highlight the emerging potential of OoC for cancer management applications. For the comprehensive review, the following sections have been discussed in the current paper respectively: What is organ-on-a-chip (OoC) technology; Tumor microenvironment; Microfluidics aided organ-on-a-chip; Organ-on-chip in cancer investigations; 2 and 3 dimensional; Bio-printing and hydrogel scaffolding in cancer cell cultures; Cancer drug sensitivity modeling by OoC; Discussion: the trend of cancer on-chip; Conclusion and future prospective.

## What is organ-on-a-chip (OoC) technology

**Fig. 1:**
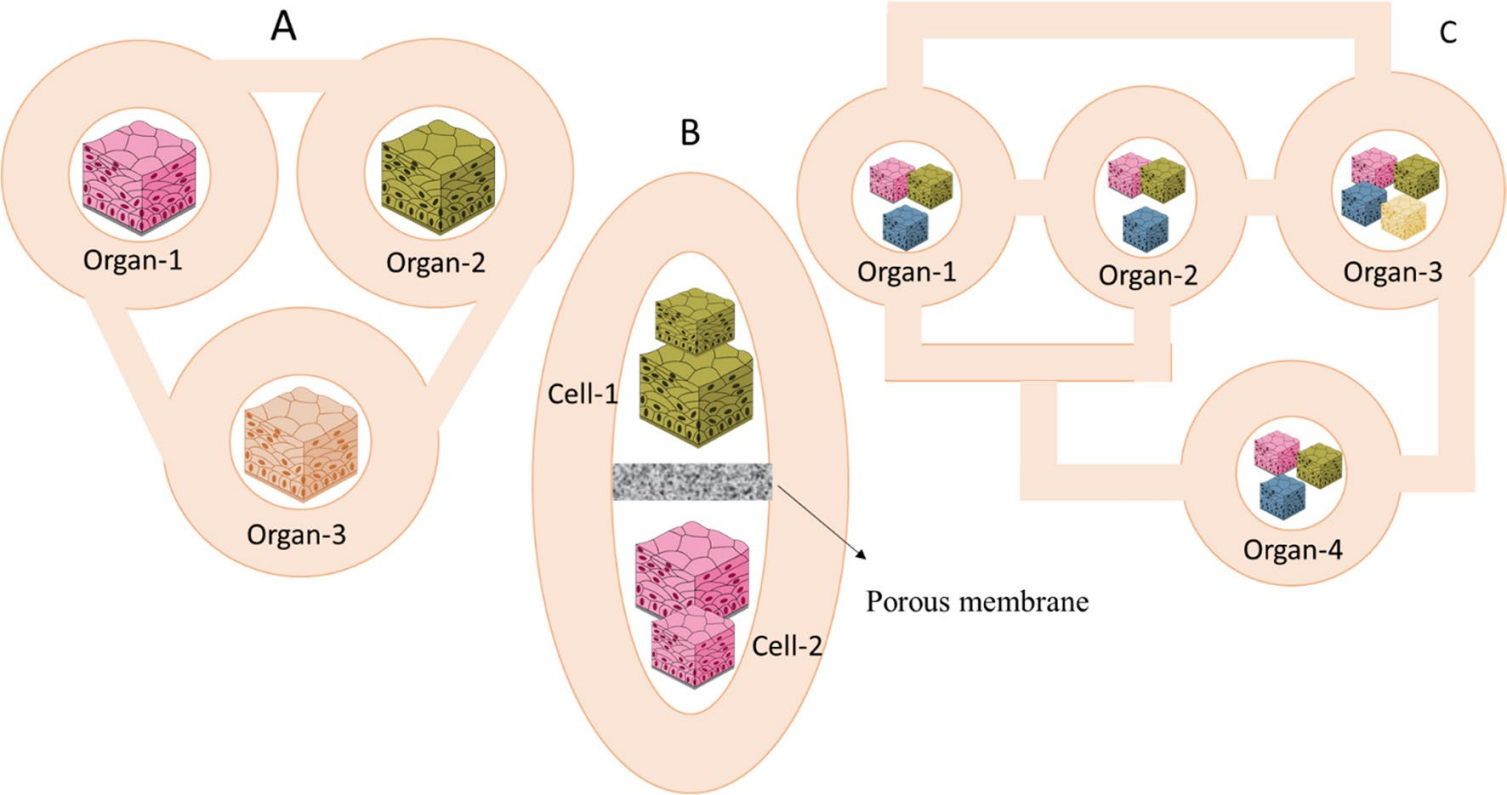
Three strategies of OoC perfusion systems: **A** This design allows for simple and direct connection of many organ modules via external channels/tubing, selectively switching flow to individual chips (on/off), or directing flow via external fluid valves can be directed. **B** The chip contains multiple fluidic chambers in this design, each housing a specific organ, tissue, or homogeneous single-cell culture. **C** This design allows the integration of relatively complex individual organ models (e.g., the liver) onto individual chips, adapted from ref [19]. **A** creates multiple simple microfluidic chambers/chips, each containing a cell monoculture connected via microchannels or tubing. This design allows for simple and direct connection of many organ modules via external channels/tubing, selectively switching flow to individual chips (on/off), or directing flow via external fluid valves can be directed [20, 21]. Additionally, modules can be connected/disconnected at desired points as well as after cell differentiation. However, the configuration of cell types in this design does not mimic the in vivo configuration, where the cell–cell interface distances (channels/tubes) between different cell types are relatively large [20, 21]. **B** Integration of multiple organs on a single chip. The chip contains multiple fluidic chambers in this design, each housing a specific organ, tissue, or homogeneous single-cell culture. Each chamber can house one or two cell types in single or multiple compartments (e.g., two loaded compartments separated by a porous membrane) that mimic specific organs [22, 23]. **C** Fluid chambers are connected by physical or biological barriers, ensuring continuous communication between organs. Simple OoC chips with 2–3 fluid compartments each can be integrated by external connections via hoses and valves. This design allows the integration of relatively complex individual organ models (e.g., the liver) onto individual chips or the integration of multiple organ modules into perfusion systems [19, 24]. High-level roadmap of OoC trials and necessities, which begins with scaling deliberations and advancements through expansion phases, surrounding device development, cellularization, perfusion and automation, and validation requirements before reaching a translational stage that qualifies specific regulations, standardization, and provides a level of ease-of-see that can lead to greater OoC adoption. A high-level roadmap of OoC trials and necessities is illustrated in Fig. 2

Organ-on-a-chip (OoC) is a modern technology in the field of biomedicine, which is increasingly expanding worldwide due to its unique features. The working basis of OoC is to mimic the behavior and complex physiological and pathological processes of different cells, organs, and even multiple organs [15, 16]. From an engineering point of view, OoC is a flow- and rate-controlled microfluidic cell culture system that mimics the physicochemical microenvironment of tissues in the human body [15, 16]. Therefore, the basic principle in the development of OoC systems to create models similar to living cells is the culture of cells originating from an individual organ in the vicinity of an environment that continuously supplies nutrients through artificial or biomimetic vessels with minimal cell surface [17, 18]. In other words, in line with cell interference and continuous maintenance of paracrine signals, OoC systems should be organized with different types of cells with appropriate tissue architecture and precise cell positioning by imitating cell plates, ducts, or more complex cell organizations, organotypic structures [17, 18]. OoC is characterized by a dynamic long-term co-culture of heterogeneous cells placed in the precise geometry of a perfused microfluidic chip, resulting in complex interactions between cells and the microenvironment. Different cell types may require different nutrients and growth factors and may not be compatible with co-culture prior to differentiation/maturation. Furthermore, the structure of cells, including polarity, organization, and interfaces, differs from organ to organ. To achieve highly integrated biological models that include multiple tissue types or multiple organs, scientists can choose from three options indicated in Fig. 1.

**Fig. 2:**
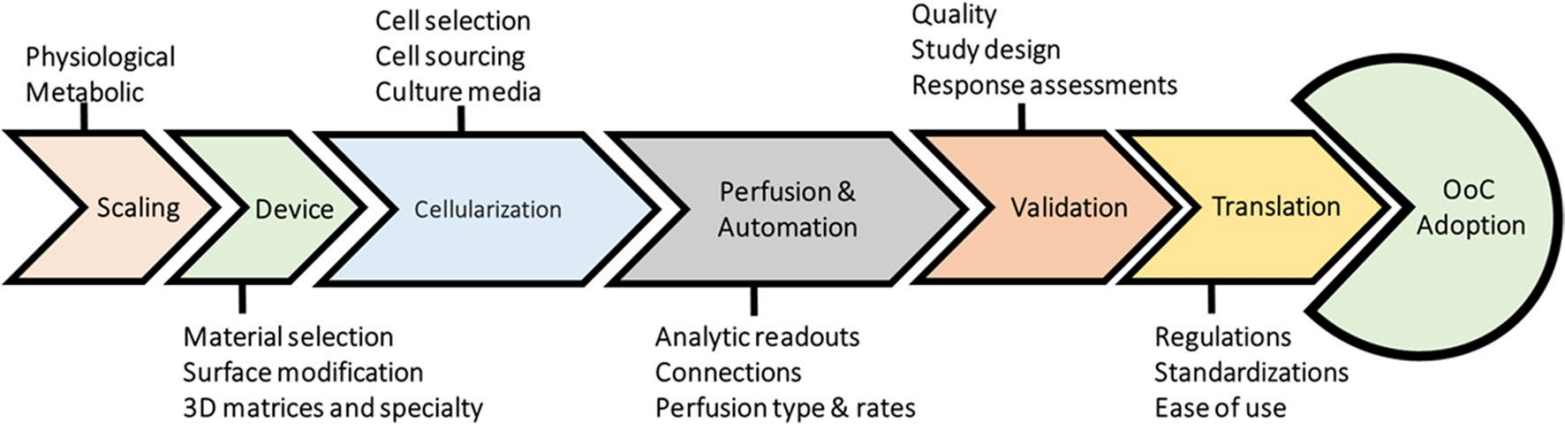
High-level roadmap of OoC trials and necessities, which begins with scaling considerations and progresses through development phases, encompassing device expansion, cellularization, perfusion and automation, and validation necessities earlier reaching a translational stage that allows specific regulations, standardization, and offers a level of ease-of-see that can lead to greater OoC approval, adapted from ref [25]

The application of OoC technology is prevalent in conducting high-throughput drug screening, studying drug absorption and metabolism, facilitating drug development and toxicology research, and creating artificial bionic microenvironments [26, 27]. OoC systems can mimic intricate tissue structures and physiological processes. The precise manipulation of biochemical and biomechanical signals in these micro-physiological systems presents the potential for cancer researchers to construct intricate representations of the tumor microenvironment. In addition, OoCs offer an engineering method for integrating cells, scaffolds, and topographical cues to generate small-scale functional tissue models with specific tissue structures [26, 27].

## Tumor microenvironment

**Fig. 3:**
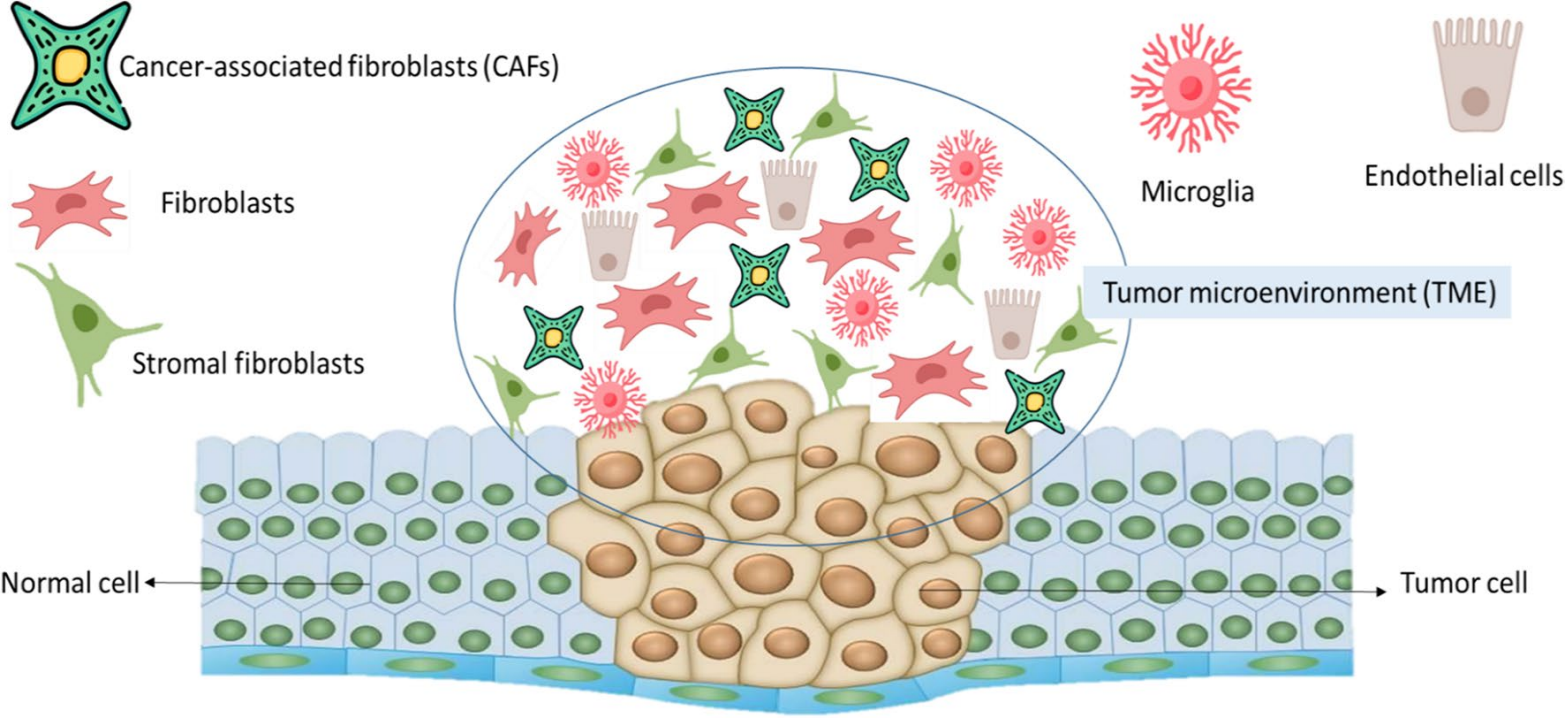
A schematic tumor microenvironment (TME). As revealed, CAFs, microglia, endothelial cells, fibroblasts, and stromal fibroblasts are the main cells in the TME

Today, in oncology, it has been proven that the complex interactions between tumor cells and their surrounding tissue, along with genetic mutations of cells, are the main causes of cancer. In other words, cancer cells present specific physical and biochemical characteristics that play a role in regulating differentiation, proliferation, invasion, cellular neoplasticity, and metastasis [28, 29]. Therefore, Tumor genetic heterogeneity poses a significant challenge to effective cancer treatment and removal. Tumor microenvironment (TME) plays a more important role in cancer initiation, progression, and drug resistance [28]. Thus, TME provides a valuable therapeutic target independent of a host of genomic uncertainties unique to each tumor. Consequently, the development of an in vitro model to understand how cancer cells interact and communicate with their surrounding tissues and how to control the progression of pathology is a vital tool for cancer research [28, 29]. TME involves endothelial cells and immune cells like macrophages, tumor cells, tumor stromal cells with stromal fibroblasts, microglia, and lymphocytes, and the non-cellular components of the extracellular matrix such as hyaluronan, collagen, fibronectin, and laminin (Fig. 3) [30].

As revealed in Fig. 3, cancer and normal cells show variances in function and morphology. In a microscopic view, cancer cells, unlike normal cells, exhibit large discrepancies in cell size and often have abnormal shapes in both the nucleus and the cells. The latter appear darker and larger because they contain excess DNA [31]. One of the main characteristics of cancer cells is that they lack certain functions, whereas normal cells function in a programmed manner. In other words, cancer cells do not respond to signals from surrounding cells and continue to grow even when they have stopped growing or have no need to grow. Cancer cells, on the other hand, escape from the immune system and grow into tumors without undergoing apoptosis [31, 32]. On the other hand, non-malignant cells in the TME play a vital role not only in the initiation of tumorigenesis but also in the stages of cancer progression and metastasis. Cancer-associated fibroblasts (CAFs) and endothelial cells are the most important primary non-malignant stromal cells of the TME. These cells have a critical and active interaction with surrounding tumor cells, with ECM, as well as with growth factors, extracellular vesicles, enzymes, secreted chemokines, and miRNAs involved in the expression of genes and proteins related to cancer metabolic pathways [32, 33]. In OoC, the dynamic microenvironment around organs, cells, and tissues is recreated on microfluidic chips. Physiologically applicable mechanical stimuli are important microenvironments that influence cellular functions and responses [34]. External syringe pumps are most commonly used to pump liquid into the microfluidic channels in a highly precise and programmed manner to provide mechanical stimulation to OoC systems [35, 36]. To minimize the size, the pump was also integrated into the microfluidic chip. Simpler delivery systems, such as passive delivery, rocking, or application of hydrostatic pressure, have also been demonstrated. A piston on the diaphragm and a pressure regulator were used to apply compressive stress to the OoC system [35, 36]. It has also been suggested and demonstrated that delivering multiple types of mechanical stimuli can better reproduce the physiologically relevant microenvironment of tissues and organs [37]. Successful manufacturing and operation of OoCs closely mimic specific physiologically applicable microenvironments, allowing definitive and rapid testing and providing an accessible, inexpensive, and easily operated platform for applied biological hypotheses. The key to the successful development of the organ-on-chip model is to focus on mimicking the organ-level pathophysiology or physiology of the observed in vivo [38]. Validation of these cancer organ-on-a-chip models requires demonstration that they effectively mimic cancer behavior and drug responses observed in vivo. There are already examples of organ-on-a-chip that mimic the cancer phenotype and response to treatment observed clinically in human patients [38]. In recent years, organ-on-chip systems, as an innovation, have provided the metastatic nature of cancer in a more realistic and better way than routine methods. These systems also summarize not only the TME but also the potential metastatic target, which are interconnected by the fluid network for metastatic modeling [39, 40]. These systems have caused the formation of a 3D cell culture system containing cell aggregations to imitate several organs in one body. In addition, these chips are a type of complex platform that helps to measure the direct effects of the reaction of one organ on another. These chips have shown great promise in the field of oncology, as they can be used to study the potential of anticancer drugs to fight human cancer cells and their effect on the tissues of multiple organs placed in a remote compartment [39, 40]. The organization of OoCs is typically derived from the structure of an organ and adapted to be easily assembled in a lab setting with the required components for normal function. OoCs, in short, are small cell culture systems created from materials that allow light to pass through, such as PDMS [11, 14]. The primary component is a microchannel that can be regulated to support the development of various types of living cells, including cell lines, primary cells, and stem cells [11, 14]. The combination of two or more cell types in the chips can create a simulated microenvironment. In separate channels, various cells, like organ-specific epithelial cells and stromal cells, are usually divided by ECM gel [16, 41]. Thus, it can reproduce the multicellular organization of human organs and interfaces between tissues, gradients of chemicals, systems for vascular perfusion, and mechanical characteristics at the level of multicellular structure [16, 41].

## Microfluidics aided organ-on-a-chip

In the past decade, microfluidic systems have been used in combination with tissue engineering methods and advanced biomaterials to develop more sophisticated systems that can successfully address the complexities of human cancer [32, 33]. These new experimental platforms have several technical and scientific advantages over traditional cancer models. Additionally, these “on-a-chip” systems can be used to study treatment-induced responses at the organ and tissue level for drug screening while also breaking down physiological complexity into more distinct units to facilitate analysis [32, 33]. All key events associated with malignant cell proliferation, survival, and metastatic spread depend on interactions between physicochemical components and the cellular of the tumor microenvironment. Due to their complexity, studies of these processes have mainly been directed using animal models [34, 35]. Recently, new “on-a-chip” cancer models have been able to reproduce the native microenvironment of human tumors in a relatively easy, accurate, and human-relevant manner, thus offering an efficient alternative to traditional in vitro and in vivo approaches. With the expansion of micro-engineered systems, numerous studies have investigated tumor cell identification, isolation, and characterization, modeling of cancer-immune interactions, mechanisms, drug screening and development, and tumor microenvironment [34, 35].

**Fig. 4:**
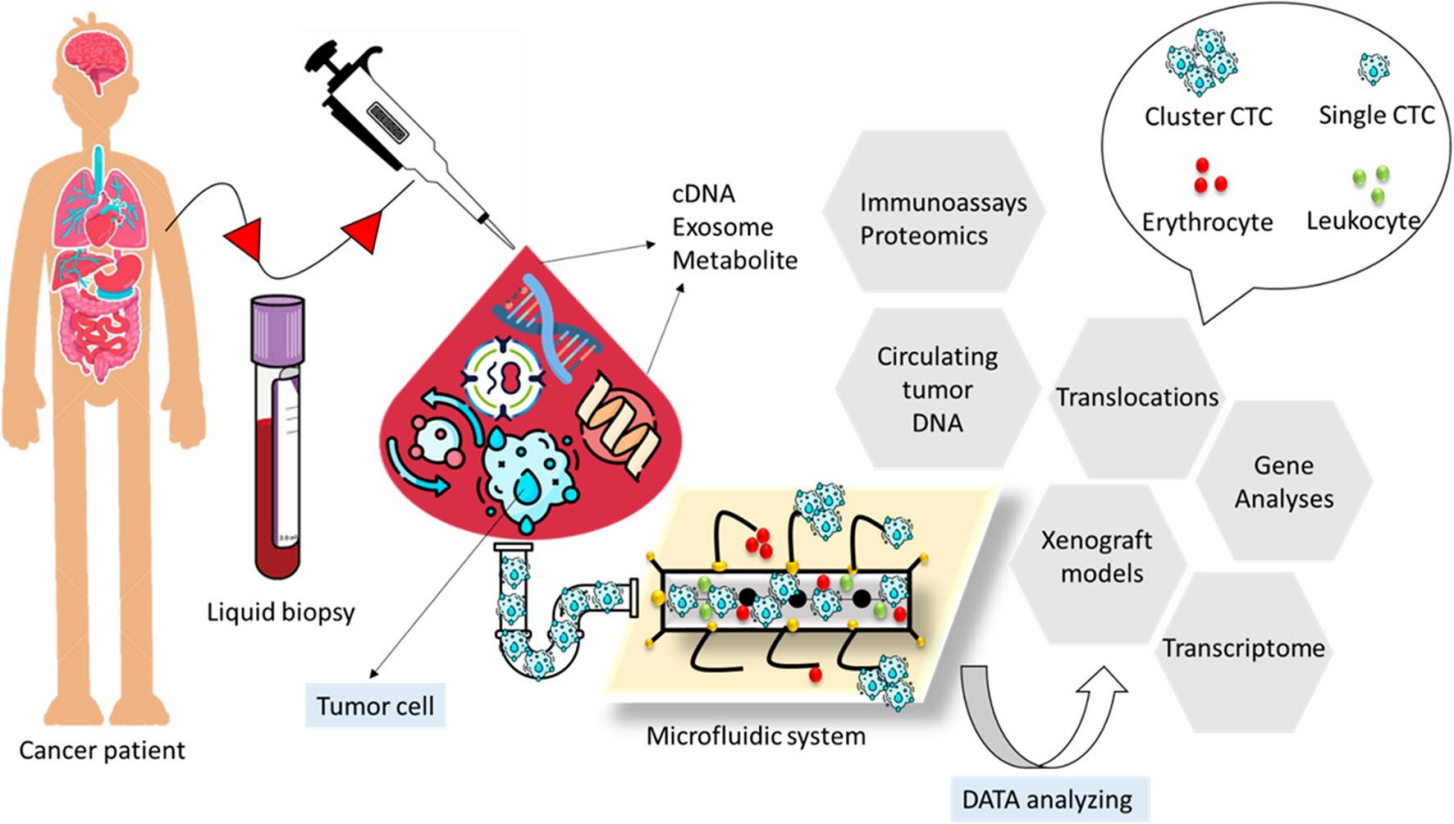
Schematic of an integrated microfluidic device for cancer cell diagnoses, different DATA analyzing methods such as immunoassay proteomics, gene analyses, and transcriptome are used in microfluidic systems, adapted from ref [42]

As shown in Fig. 4, several ml of blood is collected from a cancer patient to quantify the levels of cell-free DNA (cfDNA), exosomes, proteins, and tumor cells. Several analyses are performed regarding these biomarkers. The data obtained is evaluated to diagnose whether the patient has cancer. If the result is positive, the patient will receive treatment, and the biomarker levels will be monitored regularly. To put it simply, OoCs are small-scale fluid systems intended for simulating a more intricate physiological setting for growing and managing cells in a specific organ’s 2D or 3D structure. In addition to the unique difficulties in studying cell–cell interaction, microfluidics presents various hurdles in biomedical uses, especially in diagnostic tools where minor interactions can lead to inaccurate evaluations [43, 44].

## Organ-on-chip in cancer investigations

Organ-on-chip is a microfluidic cell culture device fabricated using microchip manufacturing technology that contains continuously flowing hollow microchannels populated with living cells arranged to simulate organ-level pathophysiology [45, 46]. By replicating the body’s multicellular architecture, chemical gradients, inter-tissue interfaces, mechanical signals, and vascular perfusion, these devices achieve tissue and organ function levels impossible with traditional 2D or 3D culture systems [45, 46]. Due to the unique advantages of “organ-on-chip” technology, its use in cancer is expanding, and some of the most recent studies will be discussed below. Cardiotoxicity is one of the most serious side effects of cancer chemotherapy. Current methods of monitoring chemotherapy-induced cardiotoxicity (CIC) and model systems to develop CIC platforms, either in vivo or in vitro, fail to detect early signs of CIC [47, 48]. Furthermore, breast cancer (BC) patients with pre-existing cardiac dysfunction may experience different CIC incidences. A cardiotoxicity-on-a-chip platform containing iPSC-derived cardiac tissue in contact with BC tissue was developed to address the lack of model systems that reproduce complex patient-specific cardiac functions to detect early symptoms of CIC. The advanced Multiplexed EC immune system aptasensors can perform noninvasive monitoring of multiple cell-secreted biomarkers [47, 48]. This platform can assess the effects of DOX on the functionality and toxicity of induced pluripotent stem cell (iPSC)-derived cardiac tissue with pre-existing cardiac fibrosis as a step toward early detection and prediction of CIC in individuals with pre-existing cardiac fibrosis which can be compared with normal heart tissue [47, 49]. To evaluate nanoparticle-based drug delivery systems, an in vitro breast tumor model was fabricated on a chip consisting of microvascular walls, extracellular matrix (ECM), and equal-sized multicellular tumor spheroids (MCTS). The created system can evaluate the efficacy and delivery of the drug transport system in T47D and BT549 spheroids, two cell lines representing triple-negative breast cancer (TNBC) and non-TNBC, correspondingly [50, 51]. This microfluidic platform allows the evaluation of dynamic transport behavior within the system and the valuation of cytotoxicity in situ [52]. A microfluidic system that controlled and simulated multi-factors of the TME for three-dimensional (3D) assessment of tumor invasion into the stroma was advanced. The finding showed that increased secretion of IL-6 among both noncancerous cells (MCF-10A or HDF-n) and cancer cells (MDA-MB-231) after co-culture contributes to cancer cell invasiveness, and this was confirmed by an IL-6 inhibitor assay. Lastly, the drug efficacy of paclitaxel was reflected in modifications in cancer cell viability, migration ability, and morphology. The result indicated that microfluidic devices could be a suitable tool to screen anti-metastatic drugs and to study the mechanism of tumor invasion into the stroma [53]. The tumor microenvironment is highly intricate and serious for tumor development and drug resistance. The endothelium is crucial in the tumor microenvironment: The endothelium supplies the tumor with nutrients and oxygen and is essential for systemic drug delivery [54]. Therefore, a simple and user-friendly microfluidic device has been developed to facilitate the co-culture of a 2D endothelial model and a 3D breast tumor model, enabling studies on drug delivery and cellular interactions [54]. The results revealed the endothelium was functional, while the tumor model showed in vivo features, preferential proliferation of cells, and oxygen gradients with better access to nutrients and oxygen [55, 56]. Using limited amounts of material, an organo-platform was developed to allow the real-time culture of 96 perfused micro tissues. In this work, the TNBC cell lines, MDA-MB-231, MDA-MB-453, and HCC1937 were designated based on their P53 and different BRCA1 status and were seeded in the platform [57]. Interestingly, biological material transport at the arterial end of capillaries in the TME was simulated by a fluidic microsystem. This study showed that static and perfused 3D microvascular networks are formed under conditions before and after loading patient tumor cells or tumor organoids into adjacent compartments. The vascular network was also found to be a physiological source of nutrients and/or drugs for tumors [58]. An innovative microfluidic system was advanced to real-time profiling of glucose concentrations and oxygen inside the device as well as ROS generation, effects on cell growth proliferation, and apoptosis. The proposed system lies between in vivo testing in preclinical drug development and traditional cell culture-based evaluation, as only compounds that show promising activity in microfluidic models proceed to in vivo testing and reduce the number of animals required for testing [59]. Novel bioprinted reconstituted glioblastoma tumors composed of patient-derived tumor cells, vascular endothelial cells, and extracellular decellularization were designed. In this work, the brain tissue matrix was collected in an organized section consisting of a concentric ring of cancer stroma by maintaining the radial oxygen gradient that has the structural, biochemical, and biophysical characteristics of typical tumors [60]. Additionally, the glioblastoma-on-a-chip duplicates clinically perceived patient-specific resistances to treatment with simultaneous temozolomide and chemoradiation. The patient-specific tumor-on-a-chip cannot only be useful for the identification of effective treatments for glioblastoma patients resistant to the standard first-line treatment but can also be used to determine drug combinations associated with superior tumor killing [60]. A 3D glioblastoma multiforme (GBM) model on a separable collected microfluidic tool could be used to study anti-GBM drug testing and for GBM aggressiveness. This method enabled examinations into the viability, proliferation, invasiveness, and phenotype transition of GBM in a 3D microenvironment and under constant stimulus by medications [61]. In order to mimic the TME ideally, a microfluidic-based 3D dynamic cell culture system was developed. In this system, a 3D biomimetic liver tumor-on-a-chip with methacryloyl gelatin (GelMA) and decellularized liver matrix (DLM) integrated vital tumor-derived and biomimetic liver-on-a-chip based on the integration of DLM components with GelMA. The results showed an improved ability to maintain cell viability and enhance hepatocyte function in contrast to GelMA [62]. Anticancer drug cytotoxicity was assessed by a constructed microfluidic system based on long-term HepG2 spheroid culture. The results demonstrated that the initial size of the spheroids influences the 5-fluorouracil (5-FU) drug effect as two important liver anticancer medications [63]. The human intestine on a chip demonstrated adequate cellular fidelity and physiological functionality of Caco-2 intestinal epithelium compared to static culture in vivo. However, the transcriptome dynamics controlling the morphogenesis and mechano-dynamic disruption of Caco-2 epithelium in micro-physiological culture are still unknown. Single-cell transcriptome analysis shows that gut-on-chip culture drives three clusters exhibiting distinct gene expression and spatial representation in the 3D epithelial layer [64]. A reproducible protocol for robust induction of intestinal morphogenesis in microfluidic intestinal-on-a-chip and transwell-embedded hybrid chips was developed [65]. This method involves culturing Caco-2 or intestinal organoid epithelial cells on a conventional setup and microfluidic platform, inducing 3D morphogenesis, and characterizing the established 3D epithelium using multiple imaging modalities [65]. In order to induce 3D morphogenesis of organoid cells and human intestinal epithelium using Caco-2 cells, an intestinal mimetic microdevice on a chip was designed. In the case of physical flow and suitable physiological conditions, this tool was able to spontaneously reconstruct the morphology of the intestinal epithelium in three dimensions in the intestine on a chip. The created system increased mucus production, creating an epithelial barrier and longitudinal connections between the host and microbes. This protocol allows the restoration of the functional microstructure of the intestine by controlling the basal fluid flow within 5 days [66]. A tumor-on-a-chip model was developed to evaluate the precise delivery of nano-medicine, and the efficacy of gradient-based drug-loaded nanoparticles was confirmed. Model validation was performed through viability studies integrated with live imaging to confirm the dose–response effects of cells exposed to CMCht/PAMAM nanoparticle gradients. This platform also allows analysis at the gene expression level, and downregulation of all genes examined (caspase-3, MMP-1, and Ki-67) was detected [67]. A human Colon Chip microfluidic device lined with patient-derived primary colonic epithelial cells was used to reproduce mucus bilayer formation and non-invasively imagine mucus accumulation in live cultures. This study demonstrates the in vitro generation of colonic mucus with a physiologically relevant bilayer structure that can be analyzed noninvasively in real time. The Colon Chip may provide an original preclinical tool to dissect the role of mucus in human intestinal homeostasis, as well as in diseases such as ulcerative colitis and cancer [68]. An innovative multi-compartment OoC platform was introduced to fluidly connect 3D ovarian cancer tissue and hepatocyte models, as well as systemic administration of cisplatin, to simultaneously study hepatotoxic effects and drug efficacy in a physiological context. Computational fluid dynamics was performed to generate capillary-like blood flow and predict the diffusion of cisplatin. After cisplatin concentration screening using a 2D/3D tissue model, cytotoxicity analyses were performed in multicompartment OoC and compared with static co-culture and dynamic single organ models [69]. In a new study, vascular control of platelet extravasation in ovarian cancer was modeled using OoC technology. This platform provides in vitro tools to study how cells and molecules act individually and in combination to influence human disease and progression [70]. However, tumor chips still encounter numerous obstacles before they can be widely utilized in practical pharmaceutical industrial and clinical applications, mainly due to the intricate physiological structure and microenvironment in vivo [71, 72]. Despite the challenges, tumor-on-a-chip platforms show promise in advancing cancer therapies. Interdisciplinary collaboration among researchers from material science, biomedical engineering, cell biology, biophysics, and oncology is crucial to collectively design and optimize tumor-on-a-chip systems for cancer research and drug discovery, eventually bringing bioinspired designs to clinical applications [71, 72]. An updated list of OoC approaches for different cancer types using state-of-the-art microfluidic strategies is demonstrated in Table 1.

**Table 1:** Developed OoC system for cancer microenvironment simulations

Cancer type	Organ-on-chip	Microfluidic device	Cell lines	Ref
Breast	Heart-BC	Health/fibrotic cardiac tissues	iPSC	[47]
Breast	BC-MCTS	MCTS; MCTS, ECM	HUVECs and BT-549 MCF-7	[52]
Breast	BC-MCF-10A or HDF-n	IL-6 cancer cells (MDA-MB-231) and non-cancerous cells (MCF-10A or HDF-n)	HUVECs, MCF10A, MDA-MB231	[53]
Breast	BC-endothelium	2D endothelium and MDA-MB-231 breast tumour cells	MDA-MB-231	[55]
Breast	P53 and BRCA1	96 microfluidic chips- ECM	MDA-MB-453, MDA-MB-231 and HCC1937	[57]
Breast	BC-NBFs-CAFs	A central microvascular chamber and two side implantation chambers	MDA-MB-231, MCF-7, Caco-2, CRC-268, NHLFs, ECFC- ECs	[58]
Glioblastoma	Colon and glioblastoma	“3D cell culture” system with injected hydrogel confined to the central chamber	U251 MG and HCT-116 cells	[59]
Glioblastoma	Glioblastoma	Brain-derived-ECM bioink	HUVECs and U-87 MG, and patient-derived GBM cells	[60]
Glioblastoma	Glioblastoma-drugs	Polydimethylsiloxane (PDMS, Sylgard 184, Dow Corning, monomer/crosslinker	U87 human astrocytoma cell line	[61]
Liver	Liver-DLM	DLM- GelMA	HepG2 cell line	[62]
Liver	Liver- 5-fluorouracil, 5-FU	3D spheroid culture- Liver- 5-fluorouracil, 5-FU	HepG2 cell line	[63]
Gut	Intestinal epitheliums	Polydimethylsiloxane (PDMS; SYLGARD 184 silicone elastomer, Dow Corning)	Caco-2 epithelium	[64]
Gut	Human intestinal epithelium	Two parallel micro-channels and an elastic polydimethylsiloxane (PDMS)-based porous membrane in the middle part	Intestinal epithelial Caco-2	[65]
Gut	Human intestinal epithelium	Human intestinal epithelium using Caco-2 cells or intestinal organoid cells	Intestinal epithelial Caco-2	[66]
Colorectal	Human intestinal epithelium	GEM-loaded CMCht/PAMAM dendrimer	HCoMECs	[67]
Colon	Colon organoids	Colon Organoids- Prostaglandin E2 (PGE2)	Cancer-derived epithelial cells	[68]
Ovarian	SKOV-3 ovarian cancer and HepG2 liver cell	Two fluidically independent compartments included the tissue culture chamber and the circulatory one	Human liver HepG2 cell line	[69]
Ovarian	Ovarian cancer cells	Microfluidic chambers (adjacent blood vessel channel and parallel tumor channel) were formed	A2780 ovarian cancer cells & human ovarian surface epithelial cells	[70]

*iPSC *induced pluripotent stem cell, *ECM* extracellular matrix, *MCTS* multicellular tumor spheroids, *TRAIL* TNF-related apoptosis-inducing ligand, *LUV* large unilamellar vesicle, *CAFs* cancer-associated fibroblasts, *NBFs* normal breast fibroblasts, *DLM* decellularized liver matrix, *GelMA* gelatin methacryloyl, *PDMS* polydimethylsiloxane, *HCoMECs* human colonic microvascular endothelial cells

## 2- and 3-dimensional bio-printing and hydrogel scaffolding in cancer cell cultures

**Fig. 5:**
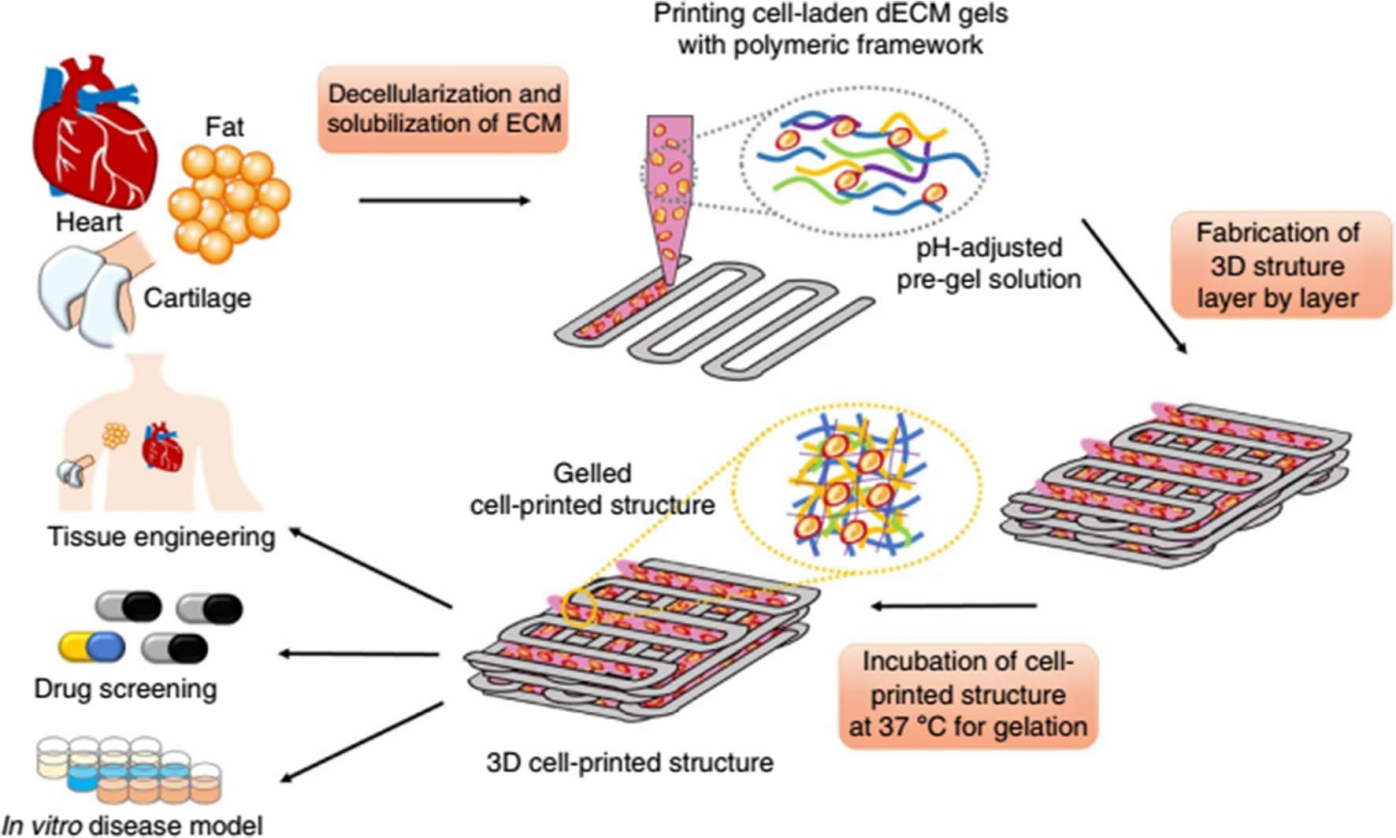
Printing 3D tissue analogues with decellularized extracellular matrix bioink. As revealed, separate tissues were decellularized later harvesting with a combination of physical, chemical, and enzymatic practices, solubilized in acidic conditions, and adjusted to physiological pH [80]

In recent years, three-dimensional (3D) cell culture techniques have become the attention of tumor cell biology research, using a variety of materials and approaches to mimic the in vivo microenvironment of cultured tumor cells in vitro [73, 74]. These 3D tumor cells exhibit many different properties compared to conventional two-dimensional (2D) cultures. 3D cell culture provides a useful platform to further characterize the biological properties of tumor cells, especially in the area of drug sensitivity, which is one of the key points in translational medicine [73, 74]. Reproducing the naturalistic biomechanical environment of cells is a prerequisite for uncovering the fundamental processes of life. The native environment is poorly replicated in 2D cell culture [75, 76]. Alternatively, although current 3D culture techniques can reproduce the properties of the extracellular matrix (ECM), it is difficult to recreate the original microenvironment. 3D organization of cells contributes to better insight into tumorigenic mechanisms in vitro cancer models [75, 76]. Applications of 3D culture include cell biology or tissue engineering studies that emphasize the presence of cells in an artificial environment. Currently, there are no models that can mimic the complexity of the natural environment in vivo [77]. In 2D cell culture, cells grow only on the surface, but in 3D culture, cells can migrate into the matrix and develop contacts in all dimensions. 3D bioprinting is one of the most promising biofabrication methods for next-generation tissue engineering [78, 79]. By combining cell-laden bio-inks with 3D printing, the construction of functional tissues and organs with complex structures from digital models is facilitated [78, 79]. After harvesting, each tissue was decellularized using a combination of physical, enzymatic, and chemical processes, solubilized in acidic conditions, and adjusted to physiological pH values, as shown in Fig. 5. Tissue printing was performed using a decellularized extracellular matrix (dECM) bioink encapsulating living stem cells in a layer-by-layer approach and then gelled at 37 °C. 3D cell-printed structures have been applied in various frontier fields, such as in vitro drug screening, tissue engineering, and cancer models.

Automated processes have the potential to scale tissue manufacturing from laboratory scale to production for drug development.

**Fig. 6:**
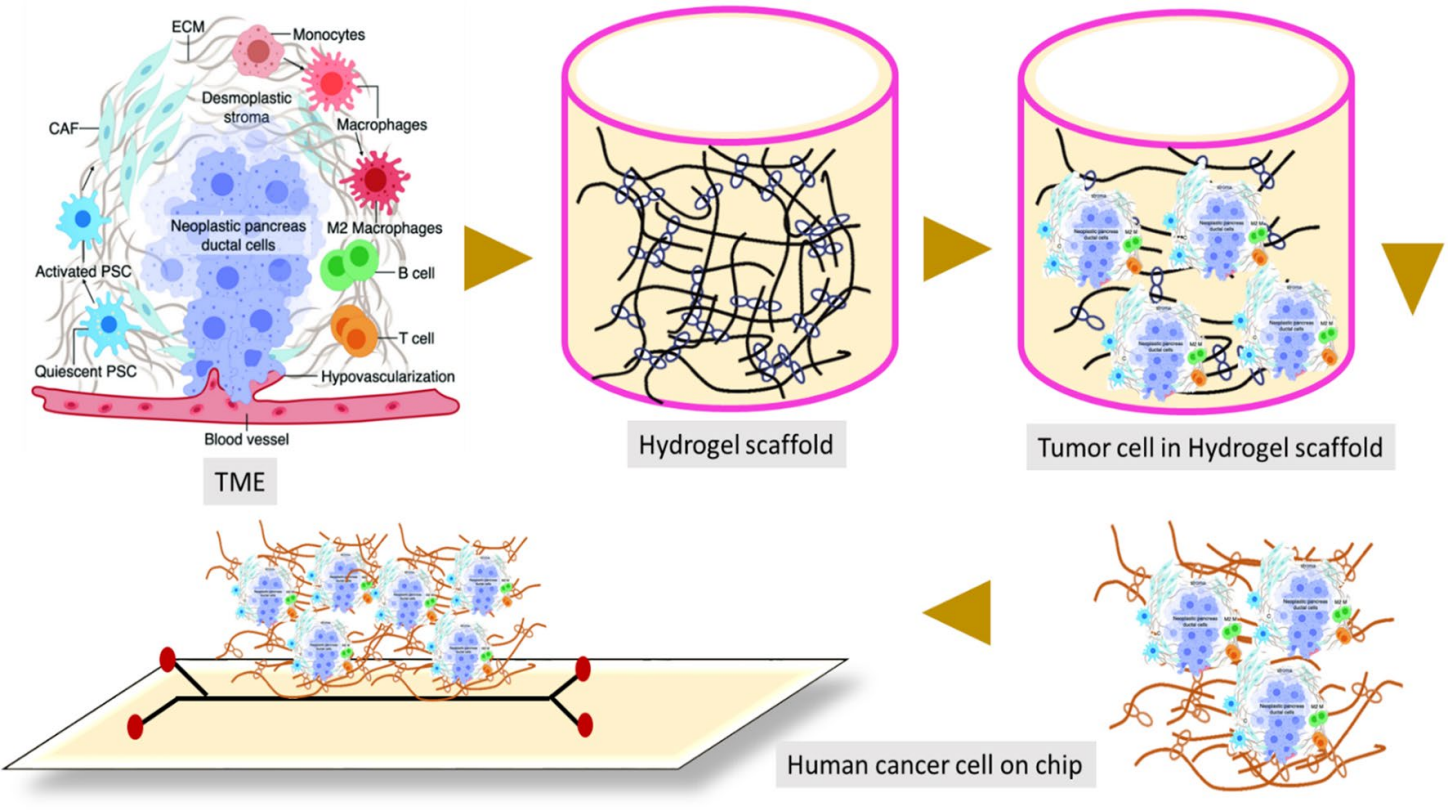
Hydrogel scaffolds in cancer cells. Scaffold- or hydrogel-based adoptive cell transfer can release mature DCs or CAR-T cells in a sustained manner and maintain the viability of cells. Functionalized scaffolds can mimic APCs to directly expand primary T cells, adapted from ref [84]

Furthermore, spatial heterogeneity of tissues can be effortlessly achieved by 3D bio-printing compared to traditional microfabrication methods. Various hydrogels have been developed to provide an optimal environment for 3D cell culture. Hydrogels are polymeric materials that form 3D networks and contain a high proportion of water [81, 82]. There are different types of hydrogels with different chemical, physical, and biological properties, such as biochemical functional groups and biodegradability. For 3D cell culture, hydrogels must have sufficient mechanical strength to promote cell immobilization, adhesion, and tissue formation [82, 83]. Additionally, hydrogels must have high porosity to transport oxygen and nutrients efficiently. Therefore, they must be sensibly optimized and selected depending on the cell type and application [83] (Fig. 6).

Despite being in its early stages, 3D cell culturing methods are starting to surpass traditional 2D cell culture methods. Additionally, each 3D culturing technique offers distinct advantages that can be utilized based on the specific experiment requirements.

Some advantages and disadvantages of 2D and 3D cell cultures, bio-printing, and hydrogel scaffold systems in OoC technology are presented in Table 2.

**Table 2:** Advantages and disadvantages of 2D, 3D cell culture, bio-printing, and hydrogel scaffold systems in OoC technology

Culture type	Advantages	Disadvantages	Ref
2D	Chip and simple, can be automated, compatible with high-throughput, easily expandable, compatible with various cell types, and standardized protocol	No ECM and TME, no concentration gradient, homogenous population, no physiological relevance, no clinically predictive	[74, 75, 85]
3D	Efficacy, drug resistance, cell–cell and cell-ECM interaction, sensitivity similar to in-vivo, co-culturing, heterogonous, inexpensive, rapid prototyping	Static environment, low TME mimicry, low resolution, low printing speed, instability for vascular channels, challenges to automate for high content screening, inefficient waste, and nutrient diffusion	[74, 75, 85]
Bio-printing	High precision and resolution, constructing highly complex tissue structures that can be automated, fine-tuning tissue architecture and size, and various bio-ink available	High expertise barrier, difficult to upscale, expensive, and need for advanced tool, difficult cell viability	[78, 79]
Hydrogel scaffold	Mimic TME, relatively chip and simple, can be automated and compatible with high-throughput. Hydrogel is available, and global application and tunable properties are available	Inefficient waste and nutrient diffusion, need for biofunctionalization, static, batch-batch variability of natural hydrogel	[81, 82]

While traditional 2D cancer models have limitations in predicting human clinical outcomes and drug responses, 3D bioprinting closely mimics the natural features of cancer in terms of morphology, composition, structure, and function [86, 87]. Applications of 3D printing have expanded far beyond department-specific patches and are most commonly used in personalized radiotherapy for treatment devices such as radiotherapy boluses and brachytherapy applicators [87]. The precise manipulation of the spatial arrangement of different cell types, vascular networks, extracellular matrix components, and 3D bioprinting facilitates the development of characteristic cancer models and, more exactly, simulating the complex interactions between cancer cells and their TME [86, 87]. Additionally, 3D bioprinting technology enables the creation of personalized cancer models using patient-derived cells and biomarkers, advancing the fields of precision medicine [86, 87].

## Cancer drug sensitivity modeling by OoC

**Fig. 7:**
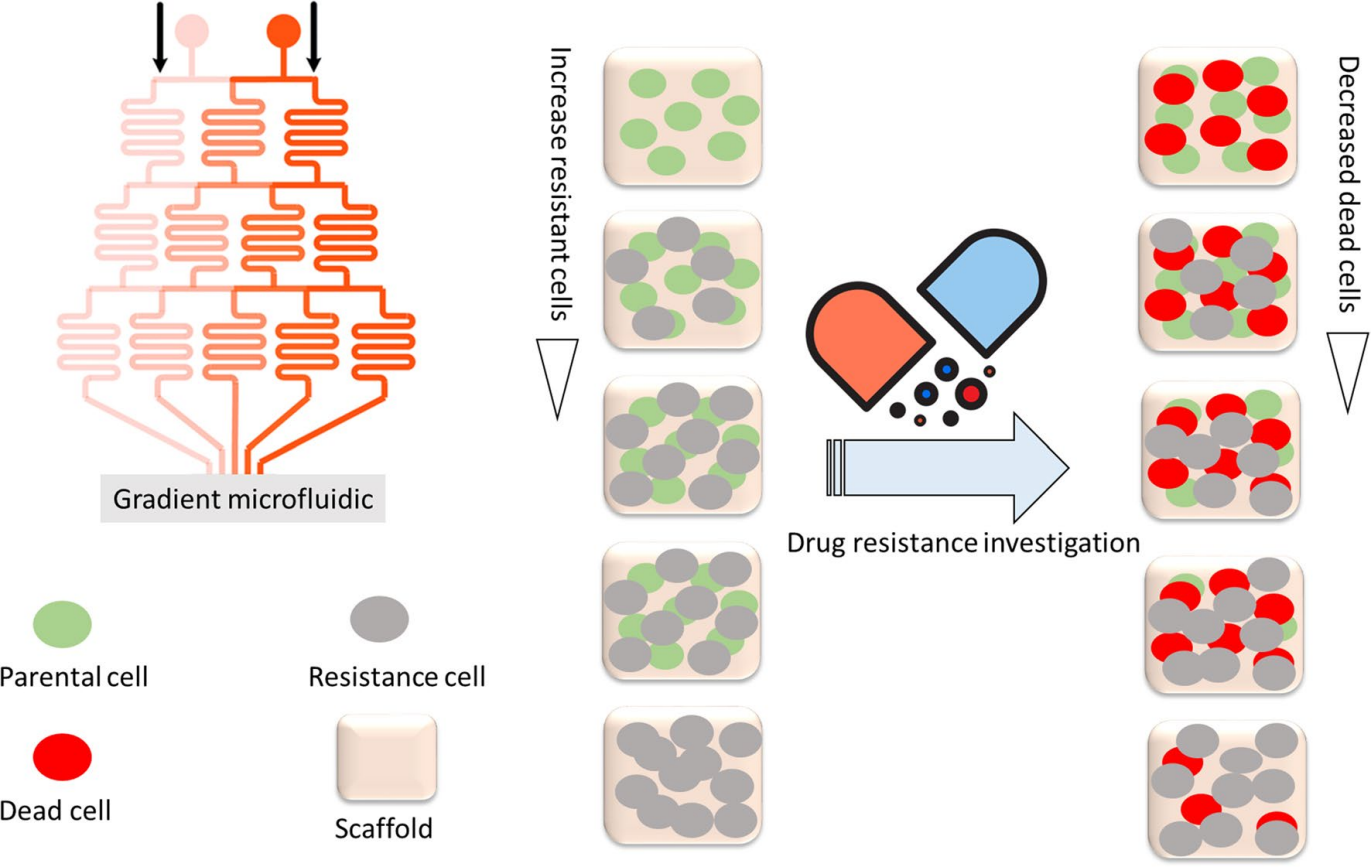
The OoC platform for creating 3D tumor models for drug resistance research, as revealed, the tumor models with varying degrees of drug-resistant phenotypes are realized by simply gradient injection of hepatoma parental and resistant cells into the microfluidic chip. This organ-on-a-chip stage will offer novel concepts for assembling 3D tumor models for drug resistance research, adapted from ref [89]

The unclear molecular mechanisms and intricate in vivo TME make it difficult to clarify the nature of cancer and develop effective treatments [88, 89]. Therefore, the development of new systems to study the role of the heterogeneous TME in individual patient responses to anticancer drugs is urgently needed and essential for the effective treatment of cancer [88, 89]. OoC platforms, which integrate 3D cell culture, tissue engineering, and microfluidics techniques, have emerged as a new method to simulate the important structural and functional structures of the TME. On the other hand, OoC technology is important for the development of new drugs [77, 89]. Because the cells in the OoC platform are directly derived from humans, species differences between humans and animals can be effectively avoided, and the toxicity of drugs to humans can be accurately assessed [77, 90]. At the same time, with the precise control and design of microfluidic chips, several types of organ-specific disease states can also be simulated in vitro, which can almost accurately reflect the dynamic change rules of drugs in the body and their effects on organs. Conduct mechanistic studies on disease pathology, efficacy of therapeutic interventions, and potential off-target side effects [90, 91]. This effectively reduces the failure rate during the clinical development stage. Additionally, OoC models are more cost-effective than traditional animal experiments. In other words, OoC technology has rapidly evolved due to the need for alternatives to animal testing and the need for real-time detection of drug toxicity [91, 92]. An OoC platform based on the inverse opal framework is planned for dynamic studies of drug resistance and tumor spheroid formation. Tumor models with varying grades of drug resistance phenotypes are achieved by simple gradient injection of hepatoma parental and resistant cells onto a microfluidic chip. This OoC platform provides a new idea for creating 3D tumor models for drug resistance research [89] (Fig. 7).

The microfluidic chip enables the creation of tumor models with different levels of drug-resistant traits by injecting hepatoma parental and resistant cells using a gradient approach. This platform for organ-on-a-chip has the potential to offer fresh insights into developing 3D tumor models for studying drug resistance [75, 90]. Furthermore, the structure and function of the heart, kidneys, lungs, and other organ systems are replicated by OoC. Tissue chips are being developed and utilized by scientists to assess the potential impacts of drugs on these tissues more quickly and efficiently compared to current methods. Get further information about these endeavors [75, 90].

## Discussion: the trend of cancer on-chip

OoC technology results from a multidisciplinary approach to life sciences, combining expertise in physics, cell biology, engineering, and chemistry [93, 94]. These models, also called microphysiological systems (MPS), aim to reproduce the biological functions of human physiology. OoC models have emerged from recent advances in microfluidics-based devices known as lab-on-a-chip [93, 94]. In other words, OoC technology aims to mimic the physical, behavioral, and structural characteristics of tissues and organs, making it a useful option in drug development. After demonstrating a culture medium representing the connection between the lungs and liver on a rectangular silicon chip, the first publication in 2004 used cells in a microfluidic device to simulate the natural biological activities of the human body [95]. Traditional experimental models to study the metastatic process, such as 3D and 2D in vitro models, lack representative complexity and cannot correctly model the TME, metastatic process, and treatment response. Therefore, microfluidics has emerged as a powerful platform for oncology research and applications due to its microscale structures that provide high-throughput screening and the ability to mimic physiological environments [96]. The focus in the field of cancer monitoring is gradually shifting from animal and 2D models to three-dimensional (3D) in vitro microfluidic and organ-on-a-chip (OoC) models that enable more complex cell replication. Microfluidics and OoC devices offer distinct advantages over outmoded tissue culture in creating a natural microenvironment for disease progress [97–99]. These allow medium perfusion that resembles the disrupted blood flow pattern, which plays an important role in platelet adhesion and EC activation during the early stages of atherosclerosis. Perfusion can also lead inflammatory cytokines into the flow, such as IL-1β (interleukin 1β), TNF-α (tumor necrosis factor-alpha), and LDL (low-density lipoprotein), further promoting the disease phenotype [97–99]. Additionally, by integrating organotypic cancer culture with microfluidic devices, “cancer-on-a-chip” systems enable the repair of the tumor microenvironment. These chips will enable a deeper understanding of cancer behavior in vivo, leading to improved preclinical evaluation of drug efficacy. By linking different physiological modules, including vascular structures, the cancer on-chip models can further study the interactions between cancer and other organs. For example, during research, a simulated 3D interface between blood vessels and cancer was used to discover the invasion of cancer cells into the bloodstream [40]. In addition, OoC platforms offer a deeper understanding of basic organ biology and can simulate the progression and development of diseases, thereby enhancing treatment and therapeutic approaches [34, 100]. The use of OoCs in basic, translational, and clinical research, such as personalized medicine and regenerative medicine, has generated considerable interest from both funding agencies and researchers [34, 100]. At present, regulatory bodies and pharmaceutical companies have taken notice of OoC’s potential as enhanced pre-clinical testing platforms [101, 102]. They could supplement current animal models and potentially take their place in the future. Animal models have traditionally served as the primary method for pre-clinical testing, with extensive data spanning many decades to support their usage. Animal models may not be considered perfect, but they remain the most effective option for pre-clinical testing apart from human testing [101, 102]. The absence of a superior model has prompted the exploration of microfluidic 3D cell cultures and OoCs as a substitute for animal testing. OoCs are preferred over animal models not only due to the significant ethical controversy surrounding breeding animals for drug testing purposes but also because they utilize human cells and simulate human organ functions, which theoretically makes them a more accurate predictive model compared to animals [103, 104]. Nevertheless, because OoCs are relatively new and lack the extensive validation required by pharmaceutical and regulatory agencies, it is important to approach them with a healthy dose of skepticism [103, 104]. In sum, the OoC technology has delivered many advantages over the routine and old methods of in vitro systems including new and more physiologically relevant ways to study and mimic the aspects of cancer, interactions, drug efficacy, and metastasis. Moreover, These models feature low fabrication costs, use human-specific stroma, allow for rapid development, are small in size for high-resolution imaging, and require minimal amounts of tissue. 

## Conclusion and future prospective

Today, in response to cancer diagnoses and treatment, OoC technology has become a promising tool in cancer research. This technology can highly reproduce the dynamic microenvironment of the tumor and other organs. With an on-chip approach, it is possible to observe and understand the mechanism and the changes taking place in metastases. In addition, multi-organ-on-chip systems enable an assessment of the impact of anti-cancer therapies (outside the human body) directly on cancer, as well as on surrounding organs, which brings new hope to personalized medicine. One of the strong points of OoC is that it offers the ability to perform synthetic biology at the tissue, cell, and organ level. Using this technology, a single micro-environmental component or drug can be used against cancer cells cultured alone or in various combinations with normal epithelial cells, endothelial cells, fibroblasts, and immune cells. It is also possible to perform multi-omics analysis and construct these chips using primary cells or induced pluripotent stem cells isolated from patients [105, 106]. In the case of tissue-like structures, compared to bioreactors, which can generate them, OoC can be a high throughput tool for generation and multi-parameter monitoring. Microfluidic platforms can replace traditional 2D/3D cell culture in vitro techniques for screening and drug development to simulate the microenvironment of tumors. Various bio-printing techniques have been introduced to enable 3D control of in vitro organ models. Hydrogels are usually prepared on supporting substrates such as films, glasses, or synthetic membranes. However, they can be combined with other materials, such as copolymers, or functionalized to create free-standing membranes. Additionally, hydrogels can be micro-fabricated by micro-structuring, photo-structuring and micro-molding, stereo-lithography, microfluidics-assisted viscous fingering, and bio-printing. The most critical point about OoC and cancer on-chip is that nowadays, they are not common methods, and other in vivo modeling methods are preferred over them. Even though OoC provides advanced biological modeling, there are major limitations that may never make them a suitable and ideal substitute for common methods. Currently, the high manufacturing and experimental implementation costs make it difficult to widely adopt organ chips, so the components need to be affordable and easily disposable. Any pricier components should be designed for reuse. For integrated system components, it is important to reduce the volume of media and the size of connectors for general use. Collecting samples from the chip could disrupt its function and cause fluctuations in the levels of different metabolites. This calls for more appropriate sensors. There is also a need for universal cell culture mediums that can be used for all organs. As the quantity of organs on the chip grows, the operations become more intricate, and the data produced carry both factual and non-translatable risks, posing a currently unsolvable challenge. When repeatedly administered over a long period or conducting on-chip studies, the biomarkers identified in vitro may not entirely represent their in vivo counterparts. In addition to the need for animal data, clinical trial results are needed to validate this technology accurately as well as to determine whether OoC models are predictive and represent human-relevant physiology across clinical outcomes and different drug classes. The main encouragement for OoC systems in cancer modeling is their potential in screening drug resistance and the development and discovery of new drugs. The main advantage of a lab-on-a-chip assay is its reduced size. By miniaturizing the total assay workflow, less sample material is needed, and the reaction and analysis times are shorter. Additionally, OoC enables researchers to assess diverse mechanisms of toxicity with greater fidelity than animal testing or conventional cell culture, lowering the risk of clinical trial attrition for drug candidates.

Although many topics were covered, the key messages from the conference can be summarized as follows: The OoC field has reached its peak, and key challenges must be addressed to avoid the possibility of disillusionment and move to the next stage of development. The fragmentation of the field is a result of intense innovation leading to resource-intensive expertise, both on the part of technology developers and end users. Overall, the lack of standardization in this area results in a multi-pronged approach that needs to be consolidated into a more focused effort. The field has reached a critical stage, and decisions made in the short term will impact the long-term success of this technology. However, there is a long way to go to reach the ideal point in OoC systems because there are still many aspects that should be explored regarding the wonderful possibilities that OoC technology offers.

## Data Availability

Not applicable.

## Abbreviations

OoC: Organ-on-a-chip
ECM: Extracellular matrix
NMs: Nanomaterials
TME: Tumor microenvironment
CAFs: Cancer-associated fibroblasts
cfDNA: Cell-free DNA
CIC: Chemotherapy-induced cardiotoxicity
BC: Breast cancer
iPSC: Induced pluripotent stem cell
MCTS: Multicellular tumor spheroids
TNBC: Triple-negative breast cancer
GBM: Glioblastoma multiforme
GelMA: Methacryloyl gelatin
DLM: Decellularized liver matrix
5-FU: 5-Fluorouracil
PDMS: Polydimethylsiloxane
dECM: Decellularized extracellular matrix
MPS: Micro physiological systems
IL-1β: Interleukin 1β
TNF-α: Tumor necrosis factor-alpha
LDL: Low-density lipoprotein
